# May Stakeholders be Involved in Design Without Informed Consent? The Case of Hidden Design

**DOI:** 10.1007/s11948-016-9811-0

**Published:** 2016-08-24

**Authors:** A. J. K. Pols

**Affiliations:** School of Innovation Sciences, IPO 1.09, Eindhoven University of Technology, 5600 MB Eindhoven, The Netherlands

**Keywords:** Ethics of design, Hidden Design, Informed consent, Procedural ethics, Research ethics, Stakeholder involvement

## Abstract

Stakeholder involvement in design is desirable from both a practical and an ethical point of view. It is difficult to do well, however, and some problems recur again and again, both of a practical nature, e.g. stakeholders acting strategically rather than openly, and of an ethical nature, e.g. power imbalances unduly affecting the outcome of the process. Hidden Design has been proposed as a method to deal with the practical problems of stakeholder involvement. It aims to do so by taking the observation of stakeholder actions, rather than the outcomes of a deliberative process, as its input. Furthermore, it hides from stakeholders the fact that a design process is taking place so that they will not behave differently than they otherwise would. Both aspects of Hidden Design have raised ethical worries. In this paper I make an ethical analysis of what it means for a design process to leave participants uninformed or deceived rather than acquiring their informed consent beforehand, and to use observation of actions rather than deliberation as input for design, using Hidden Design as a case study. This analysis is based on two sets of normative guidelines: the ethical guidelines for psychological research involving deception or uninformed participants from two professional psychological organisations, and Habermasian norms for a fair and just (deliberative) process. It supports the conclusion that stakeholder involvement in design organised in this way can be ethically acceptable, though under a number of conditions and constraints.

## Introduction

Stakeholder involvement in design is as crucial as it is tricky for both practical success and ethical justification. Concerning practical success, the more designers know about what their users actually want, the more chance they have of designing something that will actually be used. Concerning ethical justification, if a new technology affects stakeholders, those stakeholders should have a say in its design and implementation, especially if the technology or its implementation might subject them to harms, risks or unknown hazards. Besides, stakeholders may well and justifiedly protest against, oppose or boycott a technology that they feel affects them negatively and has been ‘pushed through’ without their consent. These considerations have led to the development of all kinds of design methods and innovation paradigms that promote stakeholder involvement such as constructive technology assessment (Schot and Rip 1997), co-design (Pralahad and Ramaswamy 2004), participatory design (Clement and van den Besselaar 1993; Steen 2011), value-sensitive design (Friedman et al. 2005) and responsible innovation (Stilgoe et al. 2013).

Meaningful stakeholder involvement is not easy, however. Regarding the practical side, most stakeholders are not designers themselves. Thus, their imput may not always yield *insights that are straightforwardly translatable into design specifications* (e.g. Tomico et al. 2012). They may *act strategically* to advance their interests, which may not always contribute to a socially accepted product (Blok 2014). And what stakeholders claim to desire in a product may *not always be aligned with their actual behaviour regarding that product*, for various reasons: unconscious biases, force of habit, cost-competitiveness, etc.

Regarding the ethical side, participation in the design process is difficult to get right as well. *Pre*-*set problem framings and power imbalances* are unavoidable and even necessary to start a discussion, but can exclude or marginalise stakeholder voices if not handled properly (Torgersen and Schmidt 2013; Blok 2014). The ethical requirement to be as inclusive as possible and include a diversity of voices *tends to be in tension with the practical requirement* to deliver a finished product with limited resources and within a limited time period (Bovenkerk 2012, Ch, 3; Steen 2011). And even if a technology becomes accepted by all stakeholders involved, *it does not automatically follow that it is ethically acceptable* (Manders-Huits 2010), e.g. it might violate human rights or stakeholders might have been threatened or cajoled into accepting it.

Recently ‘Hidden Design’ has been proposed as an alternative approach to stakeholder involvement in design (Afdeling Buitengewone Zaken (ABZ) 2013). The developers of this method argue that all practical shortcomings mentioned emerge from the way in which stakeholder involvement is commonly given form. In all the design methods mentioned above, stakeholder *participation* takes the form of *deliberation*. Stakeholders are interviewed, brought together in focus groups and express desires for and opinions and criticism of designer proposals. In contrast, Hidden Design claims that designers should study stakeholder participation in the form of *action*: how do they actually interact (or not interact) with a particular technology? Hidden Design’s reasons for this are similar to those of social psychologists who prefer to study human behaviour in natural settings rather than in a controlled lab. It is common knowledge in psychology that humans exhibit all sorts of biases in their reasoning and behaviour that they do not endorse consciously or may even deny having (e.g. Doris 2002). Thus, psychologists studying human behaviour cannot (only) do so by handing out questionnaires: they have to ‘hide’ experiments or present test subjects with false information on what the test is about, so they can study ‘natural’ rather than controlled, self-conscious behaviour. Hidden Design aims to do something similar: it ‘hides’ the design process, suggesting that users try out a finished product (or service) where the product is in fact continually being (re)designed on the basis of user (non-)interaction. In this way, the influence of the mere presence of the designer (and in psychological experiments, that of the researcher) on human behaviour is minimised. This helps circumvent many practical problems of stakeholder involvement. Stakeholders will not act strategically because they are not aware of having influence on a design process; only their actual behaviour, not what they claim they will do, is input for the design process; and observations of (non-)interaction are more helpful for suggesting design specifications than deliberation. The Hidden Design researchers tend to emphasize this last point with a quote from Henry Ford: “If I had asked people what they wanted, they would have said faster horses.”

Hidden Design has been developed as an answer to the *practical* problems of stakeholder involvement in design. However, so far the Hidden Design researchers have not investigated how their method scores with regard to the *ethical* aspects of stakeholder involvement. This is especially important as it is crucial to their method that subjects are unware of, or even misled about, the role they play in the design process and how data about their behaviour is being collected and used. Of course, Hidden Design is not unique in this: surreptitious data-gathering and redesign of sold products through updates (e.g. software) occurs regularly. My aim in this paper is to investigate whether, and if so, under what conditions, such design methods are ethically acceptable, using Hidden Design as a case study.

The paper is structured in the following way. In the “Hidden Design” section I explain the aim of Hidden Design and the way its design process works. In “Design Evaluated as a Psychological Experiment” I make a comparison between experiments in design and ‘hidden’ psychological experiments to investigate under which circumstances it is allowed to engage in experimental research of which subjects are unaware or deceived about. I will use the guidelines for ethical experimentation here from two professional psychological organisations: the APA (American Psychological Association) and the BPS (British Psychological Society). In “Design Evaluated as a Procedure for Settling Community Affairs” I conceptualise technology design as a way of settling community affairs and evaluate it from the angle of procedural ethics, to see to which degree observation and action can (ethically) replace a deliberative process. For this I will draw on normative guidelines from Habermas for a fair deliberative process. In the “General Conclusion and Recommendations” section I draw my conclusions and end with recommendations on how and under which circumstances stakeholder involvement organised in this way can be ethically acceptable. Most important of these are that deception should never be a first option, as it would reflect badly upon the profession of designers at large; that subjects should not be exposed to risks or harms that they do not consent to as part of everyday life; and that it can only be done if the technology’s effects are to a high degree reversible so that design decisions can be effectively and legitimately challenged by community members during the design process.

## Hidden Design

Hidden Design is a high-level framework for design: it is more concerned with the overall goals of design and its method of stakeholder involvement than with the nitty–gritty details of actual product design.^1^ The goal of Hidden Design is to achieve a particular societal goal or effect: the measure of success is ‘to what extent the lives of the people we design for change towards the project’s goal’. (ABZ 2013, p. 3). This goal implies an unusual focus of the Hidden Design method that brings it closer to persuasive technology (Fogg 2003) than to product design: where many design methods focus on designing functional *technical artefacts*, Hidden Design aims at creating particular *socio*-*technical systems* where technical functioning and human behaviour combine to achieve the desired goals or effects. At the same time, it realises that such systems (unlike technical artefacts) cannot be designed top-down, they have to emerge from a particular configuration of technology, stakeholders and institutions. What Hidden Design thus does is introduce a particular technology and (re)design it, using stakeholder interaction as its input, until a desired socio-technical system emerges.

**Fig. 1:**
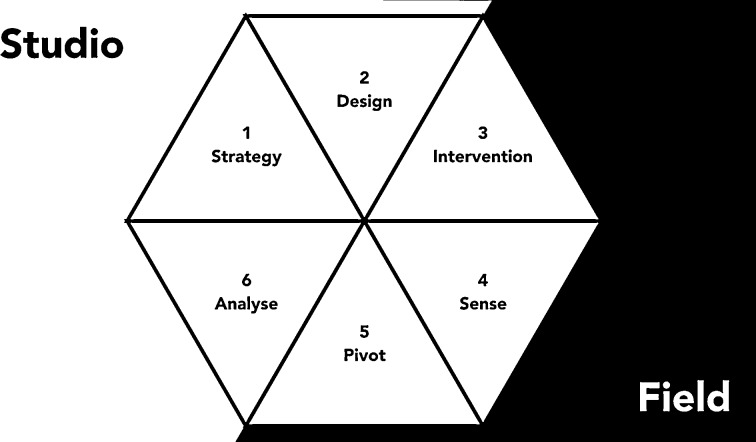
The Hidden Design method

Hidden Design consists of six steps that are applied in iteration until the desired socio-technical system has emerged or it has otherwise been decided that the design process should stop (Fig. 1). It starts with the definition of a project goal and identification of strategies that might lead to that goal (step one). From this, a technical protoype is developed together with an intervention scenario: what roles will the designers play in order to introduce their prototype in a community (step two)? The prototype is then marketed or introduced in the community by the designers as if it were a finished product or professional service in step three, the intervention stage. The method stresses the importance of making the intended users believe that they are dealing with an actual product or service, not an experiment, hence the development of an intervention scenario beforehand. This is so that the intended users will not behave strategically, try to please the designers but abandon the technology later on, or conversely, boycott the technology for reasons having nothing to do with usability. Of course, the technology *is* not a finished product: the designers continually provide the services the technology cannot yet provide and redesign the prototype ‘behind the scenes’ with the aim of gradually designing themselves out of the system (ABZ 2013). This is done by monitoring responses to the technology (step four) and quickly adapting the technology to user behaviour as needed (step five). Finally, the results of all these steps are reflected upon in step six, which leads to reconsideration of goals and strategies, starting a new cycle in the design process.

Ideally, after some time the desired socio-technical system has emerged and development of the technology has finished, at which point the designers can transfer the managerial tasks for the technology to an entrepreneur or managing institution. Several applications of Hidden Design have for example been the electrification of villages in rural India (Tomico et al. 2012) and connecting small local retailers better to their customers through digital technology in a Belgian village (ABZ 2013).

## Design Evaluated as a Psychological Experiment

As mentioned above, the Hidden Design method has not been ethically evaluated yet. This is problematic since at its core is the need to deceive potential users about the aims of the project and the status of the technology, in order to elicit ‘natural behaviour’. However, for experiments and interventions in many fields (as well as for deontological ethics) the gold standard for ethical acceptability is informed consent: if you are going to do something that affects people, then those people have to be informed *and* have to actively consent to the intervention in order to make it ethically acceptable. One prominent engineering ethics code, the US National Society of Professional Engineers’ Code of Ethics even explicitly forbids deceptive acts, though this is particularly aimed against bribery and presenting false qualifications.^2^

What does this mean for the ethical acceptability of Hidden Design? In the following two sections I evaluate Hidden Design from two perspectives. In this section, I evaluate design as a social experiment, whereas in “Design Evaluated as a Procedure for Settling Community Affairs” I evaluate it as a replacement for deliberation in procedural ethics.

Regarding design as a social experiment is helpful for two reasons. First, the introduction of new technologies in society has been conceptualised as a social experiment before, and this conceptualisation has led to practical recommendations (e.g. Jacobs et al. 2010). Second, particularly in social psychology researchers have encountered much the same problem as Hidden Design does: people will often behave differently in experimental than in natural settings, so studying natural behaviour sometimes means deceiving them with regard to the fact that an experiment is going on—or with regard to the purposes of the experiment. This has led to sets of ethical guidelines by influential organisations such as the APA (2010) and the BPS (2014) on when such experiments are acceptable. I now turn to these guidelines to see if, and if so, how they could be applied to Hidden Design.

The APA and BPS guidelines both distinguish between two kinds of situations: those where people remain *uninformed* about an experiment and its purposes, and those where they are actively *misinformed* or *deceived.* Since Hidden Design combines elements of both, I will treat each of them in turn.

### Dealing with Uninformed Participants

Much of research in social psychology as well as in sociology (e.g. ethnographic research) consists of observing people’s behaviour in particular settings. Generally, codes of ethics allow for this without requiring informed consent, if the observations are made in a public place in which people know they may be observed. The APA adds that any photos made and voices recorded may not be used for personal identification or harm (standard 8.03). It also allows observation without informed consent in a number of specific non-public circumstances (educational or organizational studies; anonymous questionnaires dealing with non-sensitive data; if otherwise permitted by local law) (standard 8.05). The BPS adds that studies based on observations in natural settings should respect the privacy and psychological wellbeing of the participants (Section 7). Extra attention is needed if people think they are not being observed, or when local cultural values may differ from those of the researchers. For this last situation, McLeod (2015) recommends asking representatives of local cultural groups beforehand whether they would expect the research to cause distress: this would be a form of getting informed consent by proxy. He also suggests more generally that research is acceptable as long as it doesn’t do anything to participants that couldn’t happen to them in real life, and places them at no greater risk of harm than they experience in real life. In other words, research that participants would consent to if they would be informed about it (hypothetical consent).

Translated to ethical guidelines for designers, this would imply that observations and interventions may be done in real life without informing participants about the design process *if* these observations and interventions are done in public places and could also happen in real-life situations, e.g. as part of a marketing or product promotion campaign. However, designers should respect the privacy and psychological wellbeing of the participants; make sure that data they collect cannot be used for personal identification or harmful purposes; and that the designers take local cultural values into account if they operate in alien settings.

Designing for different cultures, particularly developing countries, is a field of its own and requires attention to many dimensions of difference (Best and Smyth 2011; see also e.g. Macnaghten et al. 2014; Kroesen et al. 2015). I will make a few recommendations here for taking local values into account. A first step would be to approach local communities with sensitivity, attention and compassion (Rahnema 2010) which may require ‘unlearning’ of personal framings and values regarding how people should live their life (Nieusma and Riley 2010, see also the subsection “Problem Framings in Design” on dealing with problem framings.) Designers should also keep in mind the difference between *substantive* values such as privacy and autonomy, the relevance of which can be discovered during a fair and just design process, and *procedural* values, such as exclusion of particular groups from or tolerance of violence in the decision-making process, which can threaten a just and fair design process. The good news for Hidden Design is that one of the more common cultural difficulties encountered by designers is the unwillingness of stakeholders to outright criticise or ‘disappoint’ them during a formal procedure (Best and Smyth 2011), and Hidden Design evades this by offering stakeholders the opportunity to do so in a more informal, action-oriented way.

One final remark should be made regarding the risk of harm in real life, bearing in mind the case study regarding electrification of villages in rural India. It may be that designers operate in situations where the risk of harm in real life is already greater for some groups than would be morally acceptable (e.g. due to food insecurity, armed conflicts or other threats to basic human needs), or where the law permits observations and interventions that are ethically questionable. In such cases, it seems reasonable to require that designers work proactively to minimise the risk of harm for those groups through their interventions. More generally, as the BPS states in its (2009) *Code of Ethics and Conduct*, ‘no Code can replace the need for psychologists to use their professional and ethical judgment’ (p. 4) and ‘thinking is not optional’ (p. 5). There seems to be no reason why this important disclaimer would not hold for designers performing technical experiments within society as well.

### Dealing with Deceived Participants

Performing an experiment without informing participants is a different matter from actively deceiving those participants about what is going on. Unsurprisingly, psychologists have argued that the case for active deception in experiments is generally more difficult to make than that for simply not giving information.^3^ McLeod (2015) argues that there are at least three good considerations against using deception in psychological experiments: it is a violation of individuals’ rights to choose for or against participation; it is hardly a ‘healthy’ base for a professional discipline; and it can lead to distrust of professionals in that discipline. All three considerations seem applicable to design as well and should thus be taken into consideration by methods such as Hidden Design when considering deception. (This does not imply that deception is never allowed; rather that there are good reasons against implementing it that go beyond particular cases and affect design as a discipline).

For these reasons, both the APA and the BPS take a ‘no, unless’ stance with regard to deception. The APA states that deception should not occur unless it can be justified by the significant expected value of the research *and* alternative procedures without deception are not feasible (standard 8.07). The BPS agrees with the APA on these points and adds that there should be ‘an appropriate risk management and harm alleviation strategy’ (Section 7). The APA simply forbids deception if the researchers can reasonably expect that their research will cause physical pain or serious emotional distress (standard 8.07). In addition, the BPS requires an ethics review to take place of the proposed research and McLeod (2015) recommends approval by an independent expert.

Finally, all three sources stress the importance of debriefings of participants if deception is involved. McLeod (2015) gives the following arguments for this: the debriefing can take away uncertainty or misguided ideas among participants about what the researchers were about; it can give them the feeling that they have contributed to something useful; and it allows participants to withdraw their data from the study and thus reinstate their right of choice to participate to some degree (informed consent about data use). The APA adds that debriefings are a good way to check if participants have suffered no distress (standard 8.08); the BPS remarks that if hearing of the deception were to make participants angry or distressed, then the experiment should not have taken place (Section 7).

What does this mean for design? Following the recommendations by the APA and BPS would lead to a ‘no, unless’, stance, where methods such as Hidden Design would never be the first choice of stakeholder involvement, and its implementation would need justification on two grounds. The first would be that alternative design procedures are not feasible in that situation. In fact, this is what happened in the rural Indian case study, where Hidden Design was only applied after other procedures failed (Tomico et al. 2012). The second ground would be that the deception should lead to significant added value—maybe not in terms of scientific insights, though some design insights could be more broadly applicable, but rather in terms of the societal value of the new socio-technical system.

An issue left for designers to consider is that of the *distribution* of added societal value. The APA and the BPS say nothing about who should be able to benefit from those psychological insights and how this clause applies to researchers (or in our case, designers) engaging in experiments for the benefit of a private company rather than a public institution. Most theories of justice would require at least that those who bear the risks or ‘burdens’ of the research should be able to reap its benefits or get compensation for participating.^4^ Constraint on these conditions would be that designers should not engage in deception if they reasonably can expect it to result in physical pain or serious emotional distress; that they should draw up a risk management and harm mitigation strategy; and that peer review of the design plans by an independent expert from the design community is advisable.

A final consideration is that of debriefings. These might be less important in design than in a psychological experiment: taking away uncertainty or misguided ideas among the participants of what the design project was about should not be applicable, as the design experiment is always about creating a particular socio-technical system that should have become part of the everyday life of the participants at the end. Moreover, unlike psychological experiments that (ideally) start with clearly formulated hypotheses, the goals of Hidden Design are flexible and their practical implementation is partly determined by the behaviour of the participants themselves. Similarly, the resulting socio-technical system should make clear to the participants that they have contributed to something useful by (unwittingly) contributing to design research. Designers should take note that, if the design project fails in the sense that no desired socio-technical system emerges, these points remain unaddressed. Therefore, resources should be allocated in advance to making a debriefing possible in case the design project fails. Finally, debriefings could be useful to establish the right of participants to withdraw their data (recordings, videos, photos, designer notes etc.—that in any case should be anonymised or otherwise treated to exclude the possibility of personal identification or personal harm, as per the APA standard 8.03) from the research project and thus compensate for the lack of informed consent to some degree. A debriefing could also be useful for checking whether the design project has caused any distress.

In conclusion, treating design like a psychological experiment teaches us that not informing participants or even deceiving them is not necessarily morally problematic, though ethically seen, not informing always takes preference over active deception, and informed consent always takes preference over not informing. On a general level deception should never be a first choice of method and always requires justification. Also, it requires a vision of how deceptive methods will affect design as a discipline and the image of designers among the general public, as well as a strategy for compensation for not obtaining informed consent.

While direct informed consent could (allegedly) counter the practically useful effects of non-information and deception, it is clear that there are various ways in which respect for the autonomy and privacy of the participants can still be upheld if informed consent is not sought. Methods include consulting representatives of local cultural groups whether they would consent to the research (consent by proxy), a commitment to not expose participants to risks or harms that they do not already accept as part of everyday life (obtaining hypothetical consent; consent that participants would have given if they had been asked) and debriefings in which participants are asked whether they have experienced distress (if so, they should be compensated) and are offered the opportunity to withdraw their personal data from the project (obtaining informed consent about data use). A final possibility is that people may give their informed consent to goals and (kinds of) interventions through the political process: if a community democratically decides that particular interventions are allowed (e.g. the government using persuasive technologies to promote sustainable behaviour), obtaining individual informed consent about these interventions would no longer be necessary. Of course, whether this holds depends on the quality of the democratic process and whether there is indeed a clear and identifiable community: this last issue will be addressed in more detail in the subsection “Community Decision-Making”.

## Design Evaluated as a Procedure for Settling Community Affairs

In the previous section I have conceptualised design as a social experiment involving not informing or deceiving participants, and drawn a parallel between design and psychological experiments involving deception. In this section I take a different angle: conceptualising design as a procedure for settling affairs within a community.

The conceptualisation of technology as a way to settle affairs within a community is a classical one (Winner 1980): simple examples are those of installing a speed bump to discourage reckless driving, or installing a smart grid in a rural Indian village to alleviate energy poverty. The ethical advantage of such a conceptualisation is that it can draw for its evaluation on a strong tradition in ethics, namely that of procedural justice. Procedural justice is not (contrary to substantive ethical approaches) concerned with what *action* should be done or what *outcome* should be achieved, but with what *procedure* a community consisting of diverse individuals can settle its affairs in a fair and just way (e.g. Habermas 1984; 1987; 1990).^5^ Theories of procedural justice have been an inspiration for design methods such as participatory design (Steen 2011), value-sensitive design (Yetim 2011) and responsible innovation (Stilgoe et al. 2013).

Unfortunately—for our purposes, at least—procedural ethics focuses on debate and deliberation, where Hidden Design works primarily through observation and action. (Though the designers may talk to people involved, they never present themselves as designers.) This means that established norms for fair deliberation cannot straightforwardly be applied to Hidden Design, but have to be ‘translated’. As a full, philosophically grounded translation would take us too far afield in this paper, I will rather sketch a rough draft of what such a translation would look like and what it would say about Hidden Design. Particularly, this sketch looks at the ultimate aim of procedural justice and problem framings, interest groups and how to demarcate a community, and norms for the logical, dialectical and rhetorical levels of discourse.

### Problem Framings in Design

With regard to the ultimate aim of procedural justice, Habermas sees this as validating norms for action within a community, where a norm is valid when the foreseeable consequences and side-effects from acting on it could be jointly accepted by all concerned without coercion (Habermas 1999, 42). Interestingly, Hidden Design more or less subscribes to this aim, by defining their primary goals in terms so general that they would be acceptable by anyone. For example, for the electrification project in India the goal was ‘energy access for everyone’ (personal communication). However, goals can be interpreted in many different ways, and there are many different means towards each goal. Ideally, therefore, both goals and means should be discussed interchangably (Norton 2005). In practice, however, the main goal is often pre-defined and the problem pre-framed by the organising stakeholder (e.g. Bovenkerk 2012). This is practically useful for focusing the debate (and starting it in the first place) and achieving consensus, but runs the ethical risk of excluding or marginalising those with different problem framings or goal interpretations.

While Hidden Design has reflecting on and revisiting of its goals built into its method, it cannot avoid working with prototypes, which embody both a problem framing and a solution (as the speed bump and the smart grid illustrate). While this does not make the method unethical, it does mean that designers applying Hidden Design should be aware of the problem framing embodied in the prototype. This implies that they should not only pay attention to who interacts with their prototype and why, but also to participants refusing to use the prototype or boycotting it: this may be because those participants agree with the problem framing but think the solution problematic, or they may disagree with the problem framing altogether. Obviously, both situations require very different interventions. Finally, it is important for designers to keep in mind that the ultimate aim of procedural justice, validating norms for action within a community, is slightly different from the aim of Hidden Design, which is to achieve a particular societal goal or effect. Particularly, even if Hidden Design is successful in realising its aim, designers cannot assume (and should ideally check whether) all involved jointly accept the socio-technical system without coercion. (For example, participants may feel forced into using the system, or choose exclusion from the system because they feel their views have not been adequately taken into consideration, and neither would be ethically desirable.)

The issue of design always embodying a problem (and solution) framing is relevant enough to merit more treatment here. As stated before, framing is both necessary and risky. Calvert and Warren (2014) distinguish two morally relevant effects of framing: they affect the autonomy of our judgment (by making salient particular aspects of the problem) and our ideas of who can or should partake in decision-making. With regard to affecting the autonomy of our judgement, frames can shine light on hitherto neglected aspects of a problem and can structure complex and difficult issues (positive), or they can obscure morally relevant aspects (negative). In design, these effects have been investigated by the theory of mediation. For example, Verbeek (2008) investigates how obstetric ultrasound technology allows us to perceive aspects of a fetus that we couldn’t perceive before (such as the gender), but also ‘frames’ the baby as a person by making it appear as larger and more independent from the mother than it actually is. With regard to who should partake in decision-making, frames can direct our attention to parties affected by our decisions that we hadn’t taken into account before (positive), or they can stereotype or dismiss others as ‘irrational’ and thus unworthy of partaking in decision-making (negative). In design, these effects have been investigated in inclusive design (Clarkson et al. 2003), but one could also think of design for sustainability that takes into account the people who will be affected by technology design long after current users will be gone.

Calvert and Warren (2014) give a number of suggestions for minimising the chances of introducing negative framing effects. One of the most important is to focus on the practical aspects of a problem rather than the ideology that surrounds it, which many technology designers already do as a matter of course. Other suggestions include making sure that everyone can participate in the debate (or in the Hidden Design case, trying out the design.) Note that ‘equal access’ may not always be enough: there may be barriers for participation for affected groups that may require active effort on the part of the designers to remove them. Time should be taken for building mutual trust and learning from parties with different framings (this may hold between designers and users, as well between different user groups). Other options include taking time to investigate one’s own design/framing and the unstated assumptions that go into it, and confronting users with multiple designs embodying different framings.

### Interest Groups

With regard to the ultimate aim of procedural justice and interest groups, it is also noteworthy that Hidden Design can help here exactly because of its ‘hidden’ nature. Ideally, for Habermas, all participants in the deliberation share the aim of validating norms for action within a community. However, both politics and design can be influenced by interest groups that are not so much concerned with procedural justice as well as with achieving their own goals as best as possible, and that do so by ‘…pressuring or cajoling policy-makers…’ and…’lobbying, buying political advertisements, contributing funds to parties and candidates and mobilising votes…’ (Young 2001, 674). Of course, if the design process is hidden, interest groups will not be aware of it, and thus will not engage in these kinds of strategic behaviours to promote their own interests.

Hiding the design process comes with several disadvantages regarding this ultimate aim, however. One is that the deception holds equally for interest groups out for their own gain and groups or individuals that are genuinely concerned with community goals and values and would be willing to promote them even over their own self-interest—if they were to know about the design procedure. Another disadvantage is that interest groups might have very *legitimate* interests that are affected by the introduction of a new technology, in which case they *should* be involved in the design process, whether knowingly or unknowingly.

### Community Decision-Making

A second caveat with regard to applying Habermas to technology design, one that has received little attention in the literature so far, is that the Habermasian process has been drawn up for *community* decision-making. The focus here tends to be on people who are bound together by living in one physical place (a village, a country, etc.), but who might have different values or interests—apart from a common interest in the survival and flourishing of that community. On the one hand, this makes procedural ethics less applicable to innovation processes, where stakeholders might be spread across the globe (e.g. Balkema and Pols 2015). Indeed, engineers who visit a community to proclaim that an intended nuclear waste disposal site would be perfectly safe, only to travel back to their own faraway homes afterwards, may well be met with suspicion and hostility. On the other hand, communities can be bound by ties other than place, and the whole point of many innovations is to create win–win situations, in other words, to promote survival and flourishing of all the affected stakeholders. This is not to say that engineers should necessarily create ‘innovation communities’ before they can legitimately engage in deliberative exercises, though empathy and community involvement can certainly help (cf. Rahnema 2010). Rather, I will assume that if living in a common geographical location is sufficent for creating a ‘community’ to whose decision-making norms of procedural justice apply, there is no good reason why being affected by a particular innovation could also not be a common factor tying people together into a ‘community’ that can make decisions with regard to that innovation, so that it may be governed by the norms of procedural justice.

### Discourse Guidelines

Not just any stakeholder discussion will lead to a situation in which participants achieve consensus over action-guiding norms. Habermas has drawn up a number of discourse guidelines on various levels that, when followed by all, should lead to such a consensus.^6^ These norms operate on three different levels: the logical, dialectical and rhetorical level (Habermas 1990, 87–89; cf. Gilbert and Behnam 2009).

On the logical level, speakers (1) may not contradict themselves, (2) must be consistent in applying predicates to objects and (3) may not use the same expression with different meanings. Translated to action, (1) and (2) require consistency in behaviour—this is actually what Hidden Design aims to achieve by deceiving participants: if participants do not know that they are not in a ‘normal’ situation, they have no reason not to behave as they normally do, and any changes in behaviour should be the result of normal changes in beliefs, attitudes, etc. (3) Seems not applicable as there is no distinction between expression and meaning in action, though actions may of course be done (and expressions may be used) for different reasons, and questions or interviews might be needed to find them out.

On the dialectical level, speakers (1) may only assert what they really believe and (2) must provide a reason if they want to dispute a norm or proposition not currently under discussion. Condition (2) is impossible to enforce if participants are unaware of the design experiment: people may and do engage with other socio-technical systems than that the designer seeks to design, and it seems possible that this may interfere with designer plans. However, this is not a problem as long as participants do not directly disturb the design experiment without reason. Condition (1) forbids strategic behaviour, and this is again what Hidden Design seeks to accomplish by deceiving participants. However, Hidden Design’s designers make an obvious exception for themselves because of their deception.^7^ The rather cynical though not unjustified reason for this is again the claim that if the designers won’t deceive the participants (about the purpose of the experiment), the participants will likely deceive the designers (for social or strategic reasons) and no successful design will result. As I already went into the issue of deception at length in the previous section, I simply note here that it is a violation of Habermasian guidelines and leave the empirical validity of the cynical claim and the justifying power of the designers’ argument for our non-ideal world to be addressed another day.

On the rhetorical level, (1) everyone with the competence to speak and act should be allowed to take part in the discourse, (2) everyone should be able to express attitudes, desires needs and make any assertions whatsoever, and to question the assertions of others, and (3) no speaker may be prevented from exercising his or her rights through coercion. (1) and (3) should again be guaranteed through hiding the design process, though the designers should be well aware of existing social structures that may prevent legitimate stakeholders from participating; be aware of the coercion that technology itself may exhibit (e.g. Winner 1980); and strive to be as inclusive as possible in their design of the prototype so that interaction for particular user groups is not excluded from the start (Clarkson et al. 2003).

Deliberative processes have been criticised for their inclusivity being necessarily limited: those who cannot participate in deliberation, such as animals, young children and future generations cannot influence the process, even though they may have legitimate and urgent stakes in its outcome.^8^ On this point Hidden Design actually has an advantage over deliberative processes, as parties such as animals that cannot deliberate can still interact with the technology in accordance with their interests and thus provide useful input for the design process.

A difficulty, however, lies with the requirement that everyone should be able to question the assertions of others, and here the big advantage of a discourse situation comes to light. For assertions do not change the world as actions do, and where an assertion (e.g. ‘We should drill for shale gas in this municipality’, ‘We should kill the wolves that eat our sheep’) may be freely challenged, an action (actually drilling for shale gas in that municipality, or killing the wolves) may have irreversible consequences that effectively constrain the action opportunities of others, including limiting their ability to challenge or undo that action and its consequences. Indeed, technology has been conceptualised as enforcing or suggesting particular socio-political structures (Winner 1980) or a particular morality (Latour 1992) that, once in place, may selectively constrain or coerce people. There is always a trade-off here in the sense that design is both opening up and closing off options for action: fracking technology allows us to drill for shale gas while at the same time closing off the possibility for having a nature preserve at the same site. Hidden Design partly compensates for potentially irreversible effects through iterative monitoring and redesign so that changes in design can be undone if desired. However, this may not be possible for all kinds of technologies and designers should consider in advance whether their application of Hidden Design may have irreversible effects, and if so, whether they would be acceptable given the value and distribution of the potential gains, require compensation or a suitable exit strategy on the part of the designers, etc.

### Achieving Consensus

Hidden Design shares a certain optimism with Habermas, and indeed with all participative design methods that were mentioned in the introduction: all assume that following the process will eventually lead to a decision. For Habermas, this means achieving consensus on how a particular issue should be settled within a community; for Hidden Design, this means the emergence of a socio-technical system that fits its goals. Yet deliberation is not always able to solve value disagreements (such as in the debate on GM crops, see Bovenkerk 2012), and likewise it seems that Hidden Design may well run into value disagreements where no given design can appeal to two groups of stakeholders at the same time. Neither Habermas nor Hidden Design offers an ethically justified way to balance the ethical goal of inclusion against the practical aims of achieving consensus or a workable socio-technical system. Suggestions by others include accepting that consensus may not always be possible, but that by following the relevant procedure we may still attain consensus on measures/designs, if not on underlying values (Norton 2005, Ch. 4), or that we may attain a deeper understanding of the problem and each other’s viewpoints and keep the door open for future consensus (Gutmann and Thompson 1996; Swierstra and Rip 2007). Alternatively, we may adopt a two- or three-track approach, where the decision-making/design track feeds on, but is not identical to, tracks in which more fundamental problem exploration and discussion takes place (Bovenkerk 2012, Ch. 3); or design for flexibility so that, if new factual insights emerge or values change, the socio-technical system can easily be adapted (e.g. van der Velden 2009; Kiran 2012; Dechesne et al. 2013).

In case these options cannot be applied and circumstances demand a decision, a possible option is trying to achieve reflective equilibrium, where moral judgements of stakeholders are critically reflected upon with the help of moral principles and background theories (van de Poel and Zwart 2010). Another option is abandoning the aim of consensus, where everyone fully agrees with with the final solution, and allow bartering, where people may negotiate on the basis of their own self interest (“I’ll allow you X if you concede me Y”), or a compromise, where everyone jointly tries to work out a solution that is most acceptable for everyone, even though not everyone will agree on all aspects of the design (Bellamy 1999). Gilbert and Behnam (2009, 229) follow Habermas (1996) in asserting that compromises are only acceptable as a second-best solution under three conditions: (1) the compromise should be more advantageous to all participants than maintaining the status quo; (2) free-riders who are unwilling to cooperate should be excluded from the arrangement and (3) exploited parties who contribute more to the cooperative effort than they gain from it should also be excluded from the arrangement. These conditions give such a compromise an advantage over a simple majority vote (e.g. they do not allow for the exploitation of a minority). Moreover, both consensus and compromise are at an advantage over expert judgements as a solution when dealing with complex, ‘wicked’ problems (such as how to deal with energy poverty in rural India), as these kinds of problems tend to go beyond the expertise of any one specialist or designer, and are at least as much the result of value clashes as of technical challenges (Funtowicz and Ravetz 1993).

### Design as a Procedure for Settling Community Affairs: Conclusion

In conclusion it seems that the observation-and-action-oriented Hidden Design offers an interesting alternative for deliberative procedures in design. It is able to avoid some of deliberation’s practical problems such as strategic behaviour and ideological clashes due to its deceptive nature, and is more inclusive since it requires only the capability for action to participate, not the capability to deliberate. Its ethical Achilles’ heel as a procedure for settling community affairs, however, is that the actions (and designs) of some close off options for action and redesign of others. This means that the designers and those who interact first are likely to have a disproportionate impact on the design of the system compared to those who come later, that would not be justified on moral grounds. Awareness of the problem framing inherent in the design and the implementation of corrective mechanisms (see the “Problem Framings in Design” subsection) may compensate for this. Also, flexibility in design and continuous redesign can compensate for this to some degree, so it depends very much on how flexible the technology is and how irreversible the effects of its implementation are whether this is a minor or major problem.

Finally, both deliberative and action-oriented processes share a number of ethical weaknesses. One salient example is the situation that a problem framing is needed to kick off the discussion/build the prototype, yet in situations where different parties frame the problem in different ways, there is no a priori reason to start with the particular framing of the organisers/designers and their clients rather than with another. A second example is the relative optimism that consensus/a stable socio-technical system can be achieved, where in practice different groups of stakeholders may have fundamental disagreements or value conflicts that cannot be reconciled. While unfortunate, these shared challenges do mean that there are opportunities for procedural ethicists and designers to exchange insights and draw inspiration from each other’s approaches to those problems.

## General Conclusion and Recommendations

Hidden Design is an interesting attempt to circumvent some of the practical problems of deliberative stakeholder involvement, but ethical questions have been raised about its deceptive observation/action approach. In this paper I have drawn on a number of resources to ethically evaluate Hidden Design. If we regard design as similar to a psychological experiment, one of the main lessons is that it is generally acceptable to keep its experimental nature from the participants, as long as the experiment would not expose them to risks that they would not knowingly take in real-life situations. Deception should never be a first option, and its possible effects on (public perception of) designers as professionals and design as a practice should be carefully considered before implementing it.

If we regard design as a possible way of settling affairs within a community, Hidden Design has some advantages over deliberation, such as evading pressure from interest groups, a greater possible inclusiveness and using observations of actions as predictors of future actions, rather than people’s statements about what their future actions will be. One possible disadvantage is that deliberation can explore many different and mutually exclusive possibilities, whereas the actions of some may constrain the action possibilities of others, sometimes permanently. Thus, Hidden Design may not be suitable for all kinds of situations, especially where initial choices and actions will severely constrain later action possibilities. A structured overview of the measures that need to be taken to make design methods such as Hidden Design ethically acceptable can be found in Table 1.

**Table 1:** Measures to make design methods such as Hidden Design ethically acceptable

Measures	Reason
*Concerning uninformed participants*	
Are the observations made in a public place in which people know they may be observed?	Uninformed participants may not be exposed to risks, harms or privacy violations greater than those they voluntarily expose themselves to in daily life (Obtaining hypothetical consent)
Is data gathered from participants treated so that it cannot be used for personal identification or harm?	Respect for privacy
Is privacy and personal wellbeing of the participants respected?	Uninformed participants may not be exposed to risks, harms or privacy violations greater than those they voluntarily expose themselves to in daily life (Obtaining hypothetical consent)
If the project takes place in a different cultural context than that from which the researchers come, are representatives of local cultural groups consulted whether the research might cause distress?	Partly compensates for lack of informed consent (informed consent by proxy)
*Concerning deception*	
Have other design methods without deception been tried first and found unsuccessful, or otherwise judged infeasible?	Deception is hardly a ‘healthy’ base for the design field and can lead to a general mistrust in designers
Is it reasonable to expect that the experiment will lead to significant added (societal) value?	The value of the end matters for the justification of the means
Do those who run risks/harms during the experiments benefit proportionately from it?	Requirement of distributive justice
Has an appropriate risk management and harm mitigation strategy been developed?	Risks imposed on people without their consent should be minimised and any harm mitigated or compensated for by the imposing party (Obtaining hypothetical consent)
Is it reasonable to expect that the experiment will cause no physical pain or serious emotional distress?	It is impermissible for an experiment to cause physical pain or serious emotional distress
Has the proposed experiment been reviewed by an independent expert?	An impartial assessment of the (ethical) quality of the experiment helps to avoid conflicts of interest
Is a debriefing organised after the experiment has finished, regardless of whether the experiment is successful?	Taking away uncertainty or misguided ideas about the experiment; giving participants the feeling that they have contributed to something useful; check for distress among participants; allow participants to withdraw their data from the records (informed consent regarding data use)
*Concerning procedure*	
Can the values embodied in the prototype (problem framing) be justified?	Problem framing should be supported by reasons and open for challenge from stakeholders
Is the technology as inclusive as possible/are there no social or technical structures in place that exclude stakeholders from interacting with the design?	No stakeholders should be excluded from participating in the design procedure
Are the effects of interacting with the design prototype generally reversible?	Each stakeholder should be able to challenge each design decision

A final caveat: this disadvantage runs deeper than just constraining design: designers who participate in Hidden Design already presuppose that the problem they plan to tackle is *the absence of a (or presence of an undesired) socio*-*technical system*, and the solution is a *new or improved socio*-*technical system*. These presuppositions may not be shared by everyone in the community, however: some groups might not even consider there to be a problem and other groups might recognise the problem but assume that legal/social changes, not new innovations, are necessary to solve it. Indeed, it has been argued that technology designers often over-attend to technology and under-attend to process, social structures, etc., not only by virtue of their profession, but also because of the demands of investors, research projects with pre-defined ‘deliverables’ and other social structures that equate ‘success’ with ‘working product’ (Nieusma and Riley 2010). Thus, different problem framings may suggest different technological designs, but also non-technological solutions. Again, this means that people might have a wide range of reasons not to interact with a certain system, and designers would do well to be aware of that.

## Footnotes

1 It can, of course, be combined with design approaches that focus on the product design level..

2 Rule II, clause 5. See http://www.nspe.org/resources/ethics/code-ethics. Accessed 10 March 2016.

3 Not giving information is generally, but not always morally better than deception. For example, an experiment in which participants are not informed about the fact that the experimenter will cover them in poisonous spiders is more problematic than one in which the experimenter tells participants that they are interested in speed of letter recognition, while the experimenter really measures accuracy.

4 For a more in-depth discussion of issues arising in the distribution of risks and benefits, see Hansson (2003).

5 Though for those who like complications, Habermas argues that his approach can be seen as an improvement over substantive approaches founded on individual reasoning, as different individuals may reason from different (reasonable) vantage points and thus can reach different conclusions on what is the right thing to do.

6 In our non-ideal world, it may sometimes be difficult to achieve consensus. In the subsection “Achieving Consensus” I will discuss a number of second-best options that may be implemented if achieving consensus is in practice impossible and a decision (regarding design or otherwise) has to be made.

7 In contrast, it seems that deceiving participants about the fact that they are participating in a deliberative procedure for settling community affairs would be rather more difficult.

8 Indirect representation is possible, e.g. through NGOs or ombudspersons, though its normative legitimation remains difficult (Ekeli 2005).
